# Chronic recurrent multifocal osteomyelitis: experience from a single pediatric rheumatology center over the past ten years

**DOI:** 10.1186/1546-0096-12-S1-P256

**Published:** 2014-09-17

**Authors:** Serena Pastore, Giovanna Ferrara, Chiara Sandrin, Giulia Gortani, Andrea Taddio, Alberto Tommasini, Loredana Lepore

**Affiliations:** 1 University of Trieste, Institute for Maternal and Child Health IRCCS Burlo Garofolo , Trieste, Italy

## Introduction

Chronic recurrent multifocal osteomyelitis (CRMO) is a rare inflammatory disorder that primarily affects children. Its hallmark is recurring episodes of sterile osteomyelitis. The clinical presentation is insidious bone pain with or without fever. Pathogenesis is still unknown and there is no any effective treatment.

## Objectives

The aim of our study is to describe our experience with CRMO over the past ten years.

## Methods

We retrospectively evaluated patients with CRMO who had been diagnosed at or referred to Rheumatology Service of Institute for Maternal and Child Health - IRCCS Burlo Garofolo -between 2004 and 2014. History, diagnostic imaging, laboratory and histological data were obtained.

## Results

We followed seven patients diagnosed as CRMO, 6 female and 1 male. Bone pain was the leading symptom; median age of first complaint was 11 years (range 8-14 ys). The majority of bone lesions were located in the metaphyses of the long bones (10 sites, 63%), clavicle (3 sites, 19%) and pelvis (2 sites, 12%). Five patients had more than one lesion at onset. Of the latter, one patient remained with only one bone focus. Bilateral involvement was presented in two cases. The male patient also had fever and severe acne, so received diagnosis of SAPHO (synovitis, acne, pustulosis, hyperostosis, and osteitis) syndrome. Blood examination revealed slight increased eritrosedimentation rate and normal C-reactive protein in all cases. In two cases, at any disease relapse, urine analysis revealed proteinuria without other signs of renal involvement. One patient underwent renal biopsy that showed a mesangial glomerulonephritis. In all patients X-rays were suggestive of osteomyelitis. In all patients diagnosis was formalized after biopsy, except for patient with SAPHO. The biopsy showed scattered inflammatory infiltrate and leukocytes with no evidence of bacteria or malignancies in any cases. No patients responded to non-steroidal anti-inflammatory drugs (NSAIDs) therapy. All patients received corticosteroids but only two of them reached clinical remission. Of the remaining, one received methotrexate and then infliximab with no benefit so switched to bisphosphonate with partial response and 3 received bisphosphonate with good clinical response. The patient with SAPHO received Infliximab with good response. After 4 years, patient with SAPHO is still on infliximab therapy as an attempt of withdrawal provoked a flare of the disease. Four patients are clinically asymptomatic with no therapy, one patient is on bisphosphonate therapy, and one patient showed recurrent course despite biphosphonate and biological anti-TNFα therapy.

## Conclusion

The clinical course of CRMO is variable. The metaphyses of the long bones remain the more affected sites followed by clavicle and pelvis. Laboratory tests are aspecific and the biopsy is necessary, especially in cases with singular localization. In two patients we also found intermittent proteinuria, that has never been reported so far. In our opinion two hypothesis could be considered: proteinuria is a sign of renal involvement in some cases of CRMO, or proteinuria is a side effect of treatment. In fact in one patient when infliximab was withdrawn, proteinuria permanently disappeared. Bisphosphonate therapy can be of benefit to patients with relapsing symptoms. In refractory patients biologic therapy could be considered even if no controlled studies are disposable.

## Disclosure of interest

None declared.

